# Outdoor Motion Capture at Scale

**DOI:** 10.3390/s26061951

**Published:** 2026-03-20

**Authors:** Michael Zwölfer, Martin Mössner, Helge Rhodin, Werner Nachbauer

**Affiliations:** 1 Department of Sport Science, University of Innsbruck, 6020 Innsbruck, Austria; martin.moessner@uibk.ac.at (M.M.); werner.nachbauer@uibk.ac.at (W.N.); 2 Faculty of Technology, Bielefeld University, 33615 Bielefeld, Germany; helge.rhodin@uni-bielefeld.de; 3 Department of Computer Science, University of British Columbia, Vancouver, BC V6T 1Z4, Canada

**Keywords:** motion capture, outdoor, alpine skiing, pose estimation, computer vision

## Abstract

**Highlights:**

**What are the main findings?**

- An outdoor motion capture pipeline was developed for large capture volumes using pan–tilt–zoom cameras.
- Automated detection of reference points and skier-specific keypoints improved 3D reconstruction consistency compared with manual digitization.

**What are the implications of the main findings?**

- The pipeline reduces manual post-processing considerably and makes large-scale biomechanical data collection in outdoor sports feasible.
- The approach supports the creation of sport-specific datasets for biomechanics and future 3D human pose estimation models.

**Abstract:**

Capturing kinematic data in outdoor sports is challenging, as motions span large capture volumes and occur under difficult environmental conditions. Video-based approaches, particularly with pan–tilt–zoom cameras, offer a practical solution, but the extensive manual post-processing required limits their use to short sequences and few athletes. This study presents a motion capture pipeline that automates the detection of both reference points and sport-specific keypoints to overcome this limitation. The field test employed eight cameras covering a 250×80×30 m capture volume with nearly 300 reference points. Ten state-certified ski instructors performed eight standardized maneuvers. Reference points were localized through a hybrid approach combining YOLO object detection and ArUco marker identification. AlphaPose was fine-tuned on a new manually annotated dataset to detect skier-specific keypoints (e.g., skis, poles) alongside anatomical landmarks. Continuous frame-wise calibration and 3D reconstruction were performed using Direct Linear Transformation. Evaluation compared automated detections with manual annotations. Automated reference point detection achieved a mean localization error of 4.1 pixels (0.1% of 4K width) and reduced 3D segment-length variation by 23%. The skier-specific keypoint model reached 98% PCK, mAP of 0.97, and an MPJPE of 10.3 pixels while lowering 3D segment-length variation by 0.5 cm compared to manual digitization and 0.6 cm relative to a pretrained model. Replacing manual digitization with automated detection improves accuracy and facilitates kinematic data collection in large outdoor fields with many athletes and trials. The approach also enables the creation of sport-specific datasets valuable for biomechanical research and training next-generation 3D pose estimation models.

## 1. Introduction

Motion capture in outdoor environments, such as alpine skiing, is challenging due to the need to cover large capture volumes and withstand variable environmental conditions [1,2,3,4,5,6]. Commercial marker-based systems, considered the gold standard in laboratory settings, are typically impractical for field use. A few outdoor-specific marker-based solutions exist [7], but they are expensive, complex to set up, and limited to small capture volumes, and require stable environmental conditions (e.g., absence of direct sunlight, snow surface reflections, or precipitation) [8]. As a result, field studies often rely instead on sensor- or video-based motion capture. Sensor-based systems, such as inertial measurement units (IMUs) [1], are portable and unaffected by lighting or weather. However, they suffer from drift, and even when combined with GPS/GNSS [4,5], their accuracy in determining the absolute position of body segments remains limited due to sensor noise and the complexity of drift correction [2].

Video-based motion capture is therefore a widely used approach in outdoor settings [3,6,9]. These systems typically employ multi-camera setups that are synchronized and calibrated using known reference points or checkerboard-like patterns [3,6,9,10]. They provide good localization accuracy and are not susceptible to drift, making them well-suited for capturing complex motions in dynamic environments. In a recent study, Heinrich et al. [9] used four static cameras (Full-HD resolution at 100 fps) to capture a single giant slalom ski turn. Although the turn was reconstructed accurately, manually annotating the skier’s keypoints from all perspectives required dozens of hours. This highlights the substantial time required even for a short sequence in a relatively small capture volume of 30×20 m only. For larger volumes, either more cameras are needed or the cameras must be zoomed, panned, and tilted (PTZ). Spörri et al. [6] employed such a system of six PTZ cameras to capture a skier in three consecutive GS turns. This setup not only demanded the manual digitization of body keypoints but also required every visible reference point to be manually annotated and identified for each frame across all cameras. Thus, scaling such projects to include more runs or athletes quickly becomes impractical.

Recent advances in computer vision have enabled new approaches to capturing human motion [11,12,13,14,15,16,17]. These can be divided into algorithms that identify and localize anatomical landmarks in images, referred to as 2D keypoint detection [11,12,13,18], and algorithms that can directly infer the three-dimensional structure of human movement from visual data, known as 3D human pose estimation (HPE) [14,15,16,19]. 3D HPE methods [14,15,16,19] have achieved remarkable progress, particularly considering the complexity of reconstructing a 3D scene from a single camera view only. In alpine skiing, Rhodin et al. [20] were the first to use 3D HPE to reconstruct the movement of a skier. In a later study, Ostrek et al. [21] demonstrated that this approach could achieve results comparable to IMU-based systems. The robustness of 3D HPE was further improved by the introduction of temporal constraints [19] and transformer-based architectures [14]. However, achieving the accuracy required for biomechanical research remains a significant challenge, especially in domains involving complex movements such as alpine skiing. In contrast, 2D keypoint detection has already proven to be effective and reliable [17,22,23]. For example, Zwölfer et al. [22] showed that 2D keypoint detection can accurately capture skier keypoint trajectories, while Bachmann et al. [17] extended OpenPose [12] to detect not only anatomical keypoints but also sport-specific equipment such as skis and poles. Given the high workload associated with manual digitization, 2D keypoint detection presents a promising solution for automating this labor-intensive part of the post-processing workflow.

The aim of this study is to develop a 3D motion capture pipeline for outdoor sports such as alpine skiing. The pipeline integrates open-source computer vision methods to automate the detection of reference points and skier-specific keypoints, thereby replacing the labor-intensive steps of traditional video-based approaches. The pipeline is designed to operate in large capture volumes and support biomechanical studies with multiple athletes and trials.

## 2. Materials and Methods

### 2.1. Field Test Setup and Subjects

**Figure 1:**
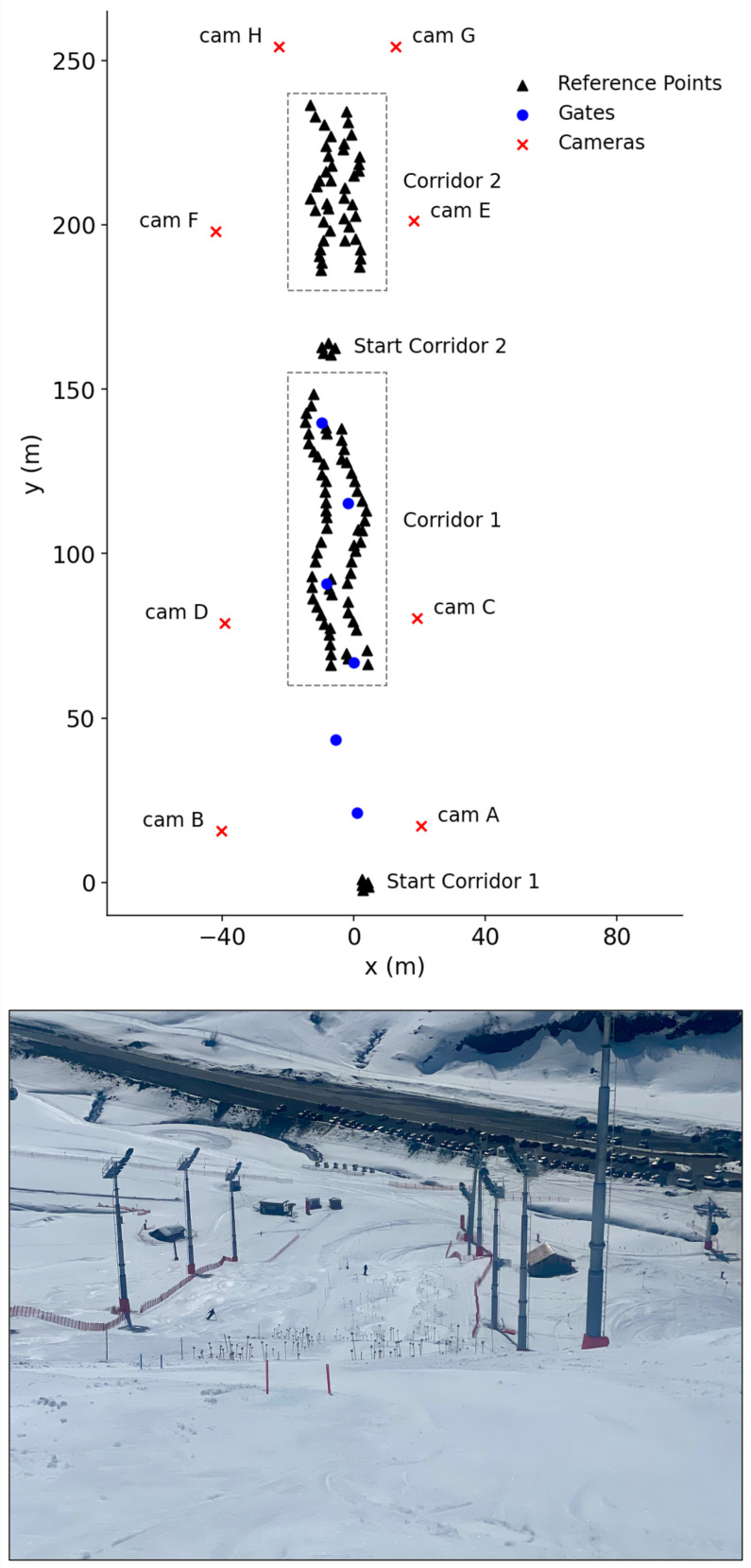
Field-test setup. Corridor 1 was set up for advanced runs (dynamic steering to carving), Corridor 2 for slower maneuvers (snowplow to parallel). Eight cameras (cam A–H; red crosses) surrounded the volume, and ∼300 reference points were installed (black triangles). Gates in the upper corridor are shown in blue. The lower panel shows a photo of the site; cameras C–F were placed on the platforms about one-third up the floodlight masts.

The experimental setup (see Figure 1) employed eight Sony AX53 camcorders (Sony Corp., Tokyo, Japan) arranged around a capture volume on a ski slope in Zürs am Arlberg, Austria. Within the capture volume, measuring 250×80×30 m, nearly 300 reference points were placed to form two distinct corridors. Corridor 1 (mean inclination $19^{\circ }$) was designed for advanced skiing techniques, including dynamic long- and short-radius turns, carving, and advanced short turns. Corridor 2 (mean inclination $12^{\circ }$) was suitable for slower skiing maneuvers, such as snowplow, snowplow-steering, and basic parallel turns in both long and short radius. Each reference point was geodetically surveyed using a Trimble S3 total station (Trimble Inc., Westminster, CO, USA) and equipped with a 9×9 cm cube displaying ArUco markers [24] on all sides. This setup enabled continuous camera calibration in every frame, accounting for panning, tilting, and zooming, which was crucial to capture the skiers at sufficient size for accurate keypoint detection. Ten state-certified Austrian ski instructors participated. The subjects (mean ± SD) had an age range of 25±4 years, a height of 171±5 cm, and a weight of 68±5 kg. Each capture session began with a warm-up period (two optional runs), followed by eight standardized runs covering all levels of the Austrian ski curriculum [25].

### 2.2. Camera Synchronization

Traditionally, genlock cameras are used in video-based motion capture to ensure simultaneous frame capture across multiple cameras [6]. Their high cost and labor-intensive setup, however, often exceed research budgets for large field tests. A common alternative is to use consumer-grade cameras at high frame rates, synchronized to a specific event, achieving a temporal offset of at most half a frame. In our experiment, Sony AX53 camcorders (Sony Corp., Tokyo, Japan) recorded at 25 fps in 4K resolution. The higher spatial detail of 4K was prioritized over frame rate, as it is crucial for keypoint detection. At ski speeds of 15 m/s, typical for carving turns, however, even a half-frame offset can introduce positional errors of up to 30 cm.

**Figure 2:**
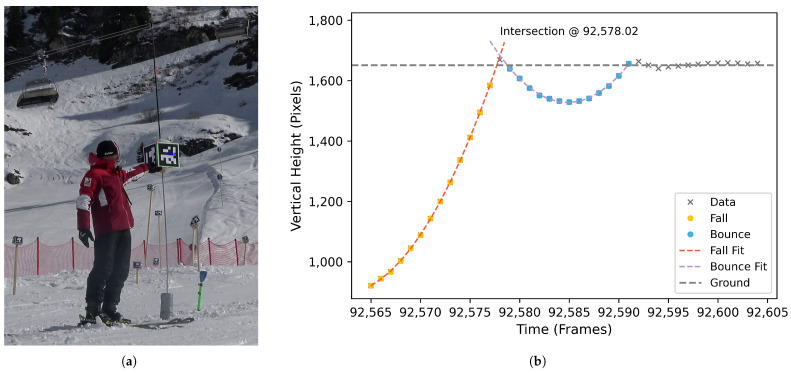
(**a**) Synchronization cube held just before release; ArUco marker detection is shown on the cube. (**b**) Temporal synchronization event: the vertical pixel coordinate of the cube is plotted against time (frames). The free-fall phase (yellow) and first bounce (blue) were each fitted with quadratic polynomials (orange, purple). Their intersection defined the synchronization event.

To address this issue, we constructed a 30×30 cm foam cube with large ArUco markers on all sides (see Figure 2a). A plastic pipe inserted through the cube allowed it to slide along a vertical carbon rod onto a damper, ensuring a straight drop without rotation. This event was recorded by all cameras. As shown in Figure 2b, the cube’s trajectory was tracked via the ArUco marker. The free-fall and first bounce were detected automatically by the trajectory’s curvature and each fitted with quadratic polynomials. Their intersection point defined the synchronization event on a sub-frame level. This procedure allowed us to detect frame offsets between cameras and interpolate both reference and keypoint data, ensuring reconstruction at the same moment. Synchronization was repeated twice per capture session to ensure reliability.

### 2.3. Reference Point Detection

To automate the detection (localization and identification) of reference points, wooden poles of lengths between 0.5 and 1.5 m were equipped with 9×9×9 cm cubes carrying ArUco markers on all sides. The cubes were large enough for reliable detection yet small enough to minimize skier occlusion. Each face of a cube displayed identical markers, while each cube was unique within the capture volume. ArUco markers were selected from the ArUco 4×4 1000 dictionary [24]. In the initial implementation, calculating the 3D positions of all marker faces from a single surveyed point on the cube was considered. Localization and identification relied directly on the ArUco detections, with the centroids of detected markers serving as position estimates. However, this proved unreliable in practice when cubes were tilted or viewed at oblique angles.

Cube localization was therefore performed using the YOLOv8 object detection algorithm [26], trained on ∼20,000 manually annotated cubes. Cube position was defined as the centroid of its bounding box, ensuring accurate localization, even when cubes were tilted or viewed from oblique angles. Identification was carried out using the OpenCV ArUco module [24]. To maximize correct detections, a grid search optimized ArUco parameters on 10 test images. A cube was considered correctly detected if it was identified by both YOLO and ArUco. Overlapping detections (>50%) were discarded.

When YOLO localized a cube but ArUco failed to provide identification—typically due to partial occlusion or motion blur—temporal information was used. Image shift vectors between frames projected previous successful detections into the current frame. If a projection aligned with a YOLO detection, the cube was accepted. This temporal matching further increased the number of correct detections.

### 2.4. Skier-Specific Keypoint Detection

Previous research identified AlphaPose [11] as a suitable keypoint detection algorithm for alpine skiing [22]. The HALPE26 body model, a 26-keypoint configuration, provides a pretrained model that detects anatomical keypoints including the head, neck, shoulders, elbows, wrists, hips, knees, ankles, and also toes and heels. To expand this model with skier-specific keypoints (ski tips, tails, and poles), ∼10,000 images from ten skiing runs (five by a female, five by a male instructor) of the present study were manually annotated using the CVAT annotation tool (CVAT, Limassol, Cyprus).

The network architecture followed the AlphaPose FastPose implementation with a ResNet-50 backbone, initialized with pretrained HALPE26 weights. Fine-tuning was performed for 200 epochs using the Adam optimizer (initial learning rate $10^{-3}$, step decay at epochs 50 and 70), a batch size of 48, and mean squared error (MSE) loss on 64 × 64 heatmaps generated from 256 × 256 input images. Data augmentation included random horizontal flipping, rotation (±40°), and scaling (±30%).

**Figure 3:**
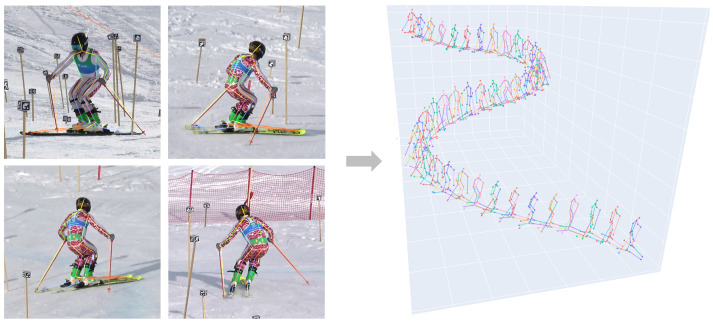
Keypoint detection and reconstruction. **Left**: Example frames from the selected run with detections from the skier-specific keypoint model. **Right**: Visualization of the reconstructed 3D keypoints for the entire run, with every fourth frame shown.

To improve generalization, the dataset was extended with images from previous experiments and the dataset by Bachmann et al. [17]. This enhanced the model’s robustness across diverse images, including skiers in recreational suits. Examples are shown in Figure 3 (left).

### 2.5. Camera Calibration and 3D Reconstruction

Camera calibration and 3D reconstruction were conducted using the Direct Linear Transformation (DLT) method [27,28]. DLT was selected due to its robustness and computational efficiency for frame-wise calibration in dynamic PTZ configurations and its established use in biomechanical field studies.

#### 2.5.1. Camera Calibration

To ensure sufficient image resolution for accurate keypoint detection, cameras were continuously panned, tilted, and zoomed. This required frame-wise camera calibration, in which a projection matrix P $\in R^{3\times 4}$ mapping 3D world coordinates to 2D image coordinates was computed for every frame and camera view. The cameras were positioned at sufficient distance from the corridor and operated in a medium to far zoom range rather than at wide-angle, so lens distortion effects were negligible for the present application. Calibration matrices were derived from reference points, either automatically detected (Section 2.3) or manually digitized for evaluation (Section 2.6). During field measurements, some reference points were displaced by participants or environmental influences (e.g., heat-induced pole tilt). To mitigate these effects, iterative outlier rejection was applied: reference points introducing large reprojection errors were removed until improvement by further removal was <5% or a minimum of six points remained. Frames with fewer than six valid reference points were excluded from calibration. To optimize calibration near the skier, only the 15 closest reference points per frame were used. The skier’s position was approximated as the centroid of the bounding box.

#### 2.5.2. 3D Reconstruction

Once calibration matrices P were derived, 3D keypoints were reconstructed. For each keypoint detected in two or more views, a system of linear equations was constructed from the 2D detections and the corresponding calibration matrices P and solved using Singular Value Decomposition (SVD), to obtain the 3D coordinates. To ensure temporal stability, the same set of cameras was used for reconstruction across all frames of a trial. Further improvements were achieved by selecting an optimized subset of cameras from the eight available perspectives by computing 3D reconstruction for all possible camera combinations and choosing the subset yielding the lowest mean reprojection error. For evaluation purposes, however, only the four manually digitized perspectives were used (see Section 2.6). Finally, reconstructed 3D keypoints were smoothed with a Butterworth low-pass filter at a 10 Hz cut-off [29]. An example reconstruction is shown in Figure 3 (right).

All computations were performed on an AIME T500 workstation (AIME GmbH, Berlin, Germany) equipped with two NVIDIA RTX 3090 GPUs (NVIDIA Corp., Santa Clara, CA, USA). Only the object detection and keypoint detection stages utilized GPU acceleration (CUDA), while calibration and 3D reconstruction were executed on CPU.

### 2.6. Evaluation Methodology

The evaluation focused on the two main contributions of our pipeline: automated reference point detection and skier-specific keypoint detection. For both components, accuracy was first assessed at the 2D pixel level, followed by an analysis of their impact on 3D reconstruction results.

#### 2.6.1. Evaluation of Skier-Specific Keypoint Detection

At the 2D pixel level, the skier-specific keypoint detection model was evaluated on the test split of our dataset using mean average precision (mAP), percentage of correct keypoints (PCK), and mean per joint position error (MPJPE), following [23].

To quantify the influence of automated keypoint detection on 3D reconstruction consistency, we first analyzed one randomly selected run not included in model training. Evaluation was restricted to a single run due to the substantial manual annotation effort required. For this run, we evaluated variations in the lengths of eight anatomical segments (lower leg, thigh, forearm, and upper arm). Segment-length variation was chosen as the primary metric because anatomical segment lengths are expected to remain constant and thus provide a measure independent of potential manual annotation error. As this evaluation was based on a single run, variation was quantified using the standard deviation of segment lengths across frames.

To extend this analysis, we also evaluated segment length variation across all 10 training runs. Mean segment lengths from manual digitization, the pretrained AlphaPose model, and our skier-specific AlphaPose model were compared. With more runs available, variation was quantified using the 95% confidence interval (CI) across run-wise mean segment lengths. In addition, statistical comparisons between methods were performed at the run level using the Wilcoxon signed-rank test to avoid treating frames as independent observations.

#### 2.6.2. Evaluationof Reference Point Detection

For the same run used to evaluate keypoint detection, reference points were manually digitized. Specifically, the 15 closest cubes to the skier in each image were annotated using the CVAT annotation tool. To evaluate the 2D accuracy of our automated reference point detection, we computed the mean pixel distance between the cube centers estimated by our algorithm and those from manual annotation. Detections with the largest deviations were visually inspected to identify potential error sources.

To assess the impact on 3D reconstruction, we compared anatomical segment length variation under two scenarios: (1) calibration with manually annotated cubes and (2) using automatically detected cubes. For this analysis, the standard deviation of each segment’s length across all frames was computed.

## 3. Results

### 3.1. Evaluationof Reference Point Detection

**Figure 4:**
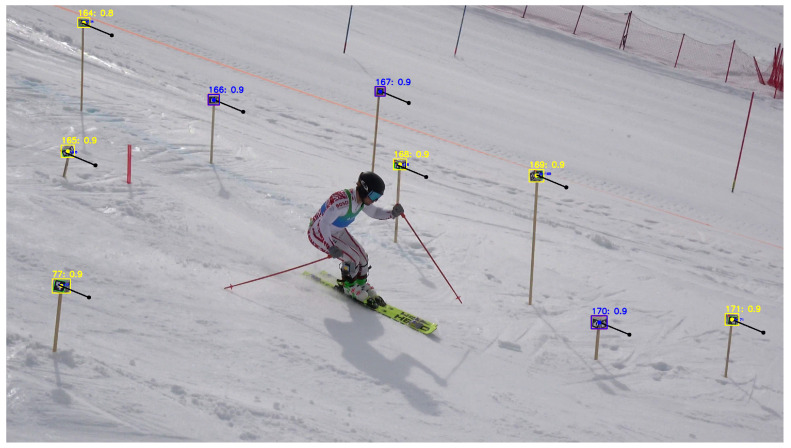
Automated reference point detection. Cubes detected by YOLO are shown with bounding boxes. Yellow indicates detections also identified by the ArUco module, while blue indicates detections not identified by ArUco but confirmed via projected detections from the previous frame (black arrows). Cube ID and YOLO confidence are denoted above each cube.

The reference point detection process is illustrated in Figure 4. Yellow boxes represent YOLO detections that were successfully identified by the ArUco module. Blue boxes indicate YOLO detections where identification by the ArUco module failed but was recovered using projections from the previous frame (black arrows).

The pixel difference between manually digitized and automatically detected reference points was calculated across all frames and camera perspectives. Out of 9600 manually annotated cubes, 48 were found to deviate more than 50 pixels. Visual inspection revealed that these were annotation errors, which were corrected. The mean pixel difference between manually digitized and automatically detected cube centers was 3.4 pixels (cam A), 3.5 pixels (cam B), 3.9 pixels (cam C) and 5.4 pixels (cam D), at an image resolution of 3840×2160 pixels. The overall average pixel error was 4.1 pixels.

To evaluate the impact on 3D reconstruction, variation in anatomical segment lengths was calculated as the mean standard deviation across all body segments (Table 1). Using manually digitized reference points, segment length variation was 4.4 cm. Using automatically detected reference points, this variation decreased to 3.4 cm, corresponding to a 23% reduction.

### 3.2. Evaluation of Skier-Specific Keypoint Detection

First, we evaluated our skier-specific keypoint detection model on the test set of our skier-specific dataset, comprising approximately 2000 images not included in training. At the 2D image level, the model achieved a percentage of correct keypoints (PCK) of 98%, a mean average precision (mAP) of 0.97, and a mean per joint position error (MPJPE) of 10.3 pixels. Visual inspection confirmed these metrics, with only a few misdetected keypoints. Misdetections typically occurred at ski tails or poles, especially under occlusion. To illustrate the model’s performance, four example images are shown in Figure 3 (left). The corresponding 3D reconstruction of the full run is shown on the right in Figure 3.

Second, we analyzed segment length variation in one manually annotated run not used for training. Quantitative results showed a mean standard deviation of 3.9 cm for manual annotations (Table 1). The pretrained AlphaPose HALPE26 model produced a slightly higher deviation of 4.0 cm, whereas the skier-specific model reduced this deviation to 3.4 cm.

**Figure 5:**
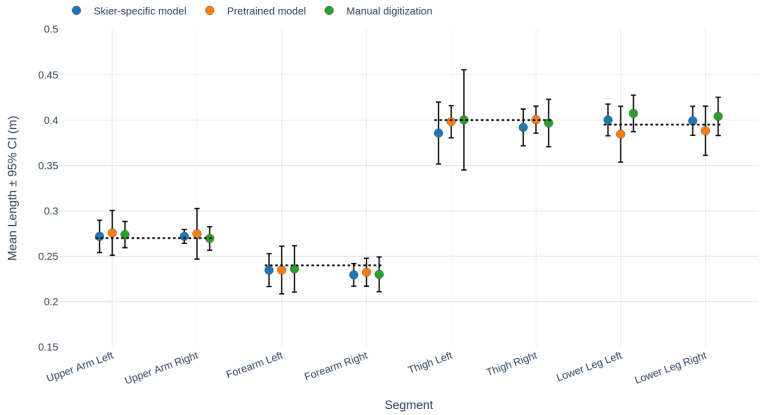
Segment length comparison, male subject. Results of three keypoint detection methods: skier-specific AlphaPose model (blue), pretrained AlphaPose model (orange), and manual digitization (green). Data points represent mean segment lengths, with error bars showing the 95% CI. The black dotted line indicates measured values from the field test.

**Figure 6:**
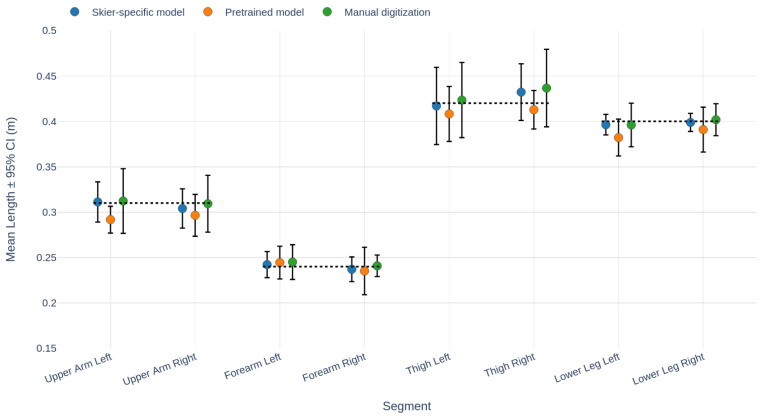
Segment length comparison, female subject. Results of three keypoint detection methods: skier-specific AlphaPose model (blue), pretrained AlphaPose model (orange), and manual digitization (green). Data points represent mean segment lengths, with error bars showing the 95% CI. The black dotted line indicates measured values from the field test.

Third, we assessed segment length variation across all 10 runs with manual annotations available. As these runs were performed by one female and one male instructor, results are presented separately (Figure 5 and Figure 6). The plots compare three methods: manual digitization (green), the pretrained AlphaPose model (orange) and our skier-specific AlphaPose model (blue), alongside measured ground-truth segment lengths from the field test (black dotted line). For both subjects, the reconstructed segment lengths closely matched the true measured values. Averaging across both subjects and all segments, the 95% confidence interval (CI) of segment length variation was 2.6 cm for manual digitization, 2.3 cm for the pretrained AlphaPose model, and 1.9 cm for the skier-specific model. Compared to manual digitization, the skier-specific model reduced segment length variation by about one quarter. Statistical analysis at the run level confirmed that the skier-specific model produced significantly lower segment-length variation than both the pretrained model (p<0.001) and manual digitization (p<0.01, Wilcoxon signed-rank test).

## 4. Discussion

In this study, we introduced a novel motion capture method for outdoor sports such as alpine skiing. By combining the advantages of video-based motion capture with the efficiency of computer vision algorithms, our pipeline reduces the reliance on manual digitization and improves the practicality of large-scale motion studies in outdoor environments.

### 4.1. Overall Performance of the Motion Capture Pipeline

The proposed pipeline achieved reliable 3D reconstructions with a mean segment length variation of 3.4 cm. This magnitude is comparable to traditional multi-camera photogrammetric systems in alpine skiing, which report 3D reconstruction errors of approximately 2–3 cm [3], and to wearable GNSS+IMU approaches reporting center-of-mass accuracies of approximately 8 cm [1]. Although these systems differ in methodology and evaluation metrics, the present results fall within the same order of magnitude as established outdoor motion capture approaches. Importantly, our result was obtained in a capture volume exceeding 200 m in length, which poses substantially greater challenges than the smaller capture volumes analyzed in previous studies [9]. Nevertheless, a mean variation of 3–4 cm may be limiting for applications requiring very high joint-level precision. Further improvements are likely achievable by incorporating more advanced filtering as well as temporal and kinematic constraints [14,19,23,30].

A key advantage of the presented approach lies in the substantial reduction in manual workload. Traditional outdoor photogrammetric systems require extensive manual digitization of anatomical landmarks, often exceeding dozens of working hours for a single giant slalom turn [9,31], which effectively limits systematic data collection across multiple athletes and trials. In contrast, processing time per run was approximately 5–10 min on the described hardware, depending on trial length. Furthermore, the entire pipeline was built on open-source models and implemented using consumer-grade camcorders, again reducing practical and financial barriers for large-scale outdoor motion capture studies.

The scalability of the proposed pipeline enables the efficient generation of large, sport-specific datasets, which provide a valuable basis for both biomechanical analyses and the further development of computer vision methods. In particular, such datasets could serve as training material for direct 3D human pose estimation models, including transformer-based approaches (e.g., 4D Humans [14]), which rely on extensive annotated data. In this sense, our pipeline not only advances large-scale outdoor motion capture today but also contributes to the foundation for future fully automated solutions.

### 4.2. Evaluation of Keypoint Detection

At the 2D image level, the skier-specific keypoint detection model achieved 98% PCK, a mAP of 0.97, and an MPJPE of 10.3 pixels. These results are comparable to manual digitization and align with previous reports of >90% PCK and ∼10 pixel MPJPE for skiing maneuvers [17,22,23]. In line with Bachmann et al. [17], we extended a pretrained body model to include skier-specific keypoints (ski tips, tails, and poles) using transfer learning.

In the 3D reconstruction, the skier-specific model reduced segment length variability by 0.5 cm compared to manual digitization and by 0.6 cm compared to the pretrained AlphaPose model. This improvement likely reflects the complementary strengths and limitations of the two training sources. Manual annotations provide sport-specific detail (e.g., ankle positions on ski boots), but their precision may vary across frames and annotators. In contrast, pretrained models offer consistent and robust priors for general body landmarks, yet they often lack sport-specific accuracy (e.g., being trained on street shoes rather than ski boots). By combining both sources, the skier-specific model consistently applies reliable priors while capturing domain-specific information essential for alpine skiing—ultimately reducing variability in reconstructed segment lengths.

The evaluation across ten additional trials (Figure 5 and Figure 6) showed the same pattern: the skier-specific model produced significantly less segment-length variation than both manual digitization and the pretrained AlphaPose model. These results must be interpreted with caution, as the runs formed part of the training dataset and may therefore bias the outcome in favor of the keypoint model. However, together with the independently annotated run that was excluded from training—our main evaluation case described above—these results also support the conclusion that the skier-specific model improves robustness and accuracy by leveraging both manual annotations and pretrained detection.

### 4.3. Evaluation of Reference Point Detection

Reference point detection proved highly reliable. The mean pixel difference between automatic and manual annotations was 4.1 pixels, corresponding to about 0.1% of the 4K image width. Given that a skier typically occupied ∼1.5 m in height, corresponding to ∼1080 pixels in the image, this error translates to approximately 0.6 cm in real-world scale. Outliers exceeding 50 pixels were only attributable to manual labeling errors, underlining the robustness of the automated approach.

When applied to 3D reconstruction, these differences became more apparent. Calibration with automatically detected reference points reduced mean segment-length variation from 4.4 cm (manual) to 3.4 cm, a 23% improvement. This demonstrates that automated detection not only eliminates labor-intensive manual work but can also improve consistency by reducing variability introduced through human annotation errors.

## 5. Limitations

First, reference points were observed to shift during the experiment due to heat and sunlight, with position differences of up to 16 cm between morning and afternoon measurements. While the outlier rejection procedure excluded strongly displaced points, smaller shifts may have contributed to the error in 3D reconstruction. Future field studies should therefore give particular attention to the stability of reference point placement under varying weather conditions.

Second, as with all markerless methods, occlusions remain a challenge. Although the multi-view camera configuration mitigates the impact of partial occlusions, snow spray frequently obscures skis and lower extremities, complicating reliable keypoint detection and subsequent reconstruction. Data were collected on two different days with varying weather and lighting conditions, and the pipeline performed robustly across both sessions. Nevertheless, broader environmental testing should be conducted in future work.

Third, our accuracy analysis was limited to anatomical segment lengths. While this provides a robust measure of consistency for body landmarks, it does not capture errors in equipment-related keypoints (e.g., skis, poles), which are equally relevant for biomechanical applications in skiing.

## 6. Conclusions

This study presents a motion capture pipeline tailored for outdoor sports such as alpine skiing, addressing the challenges of large capture volumes, moving cameras, synchronization, and the substantial manual workload in post-processing. By automating the detection of both reference points and skier-specific keypoints, the pipeline produced reconstructions that outperformed manual labeling at both the 2D and 3D levels.

The method makes large-scale outdoor motion capture feasible by enabling data collection across many athletes and trials within capture volumes of several hundred meters, providing a practical foundation for research projects in such settings where resources are often limited.

Beyond feasibility, the approach supports the generation of large, sport-specific datasets that are valuable not only for biomechanical analyses but also for training future direct 3D human pose estimation models.

## Figures and Tables

**Table 1:** Variation in segment length (cm) for different combinations of reference point and keypoint detection methods. Values represent the standard deviation of segment lengths across frames for one randomly selected trial excluded from training; overall values are averaged across all segments.

Method		U. Arm		F. Arm		Thigh		Shank		Overall
**Ref. Points**	**Keypoints**	**L**	**R**	**L**	**R**	**L**	**R**	**L**	**R**	
Manual	Manual	4.8	3.3	7.3	4.7	6.1	5.1	5.3	6.0	6.1
Manual	Automatic	4.3	3.8	3.3	3.4	5.3	4.6	3.4	3.2	4.4
Automatic	Manual	3.1	2.6	4.0	4.2	3.8	4.0	3.0	3.3	3.9
Automatic	Automatic	2.6	2.8	2.6	2.1	4.1	3.6	2.9	3.1	3.4

## Data Availability

The datasets generated and/or analyzed during the current study are available from the corresponding author upon reasonable request.
